# Matrix Topography Regulates Synaptic Transmission at the Neuromuscular Junction

**DOI:** 10.1002/advs.201801521

**Published:** 2019-01-17

**Authors:** Eunkyung Ko, Seung Jung Yu, Gelson J. Pagan‐Diaz, Ziad Mahmassani, Marni D. Boppart, Sung Gap Im, Rashid Bashir, Hyunjoon Kong

**Affiliations:** ^1^ Department of Bioengineering University of Illinois at Urbana–Champaign Urbana IL 61801 USA; ^2^ Department of Bioengineering Micro and Nanotechnology Laboratory University of Illinois at Urbana–Champaign Urbana IL 61801 USA; ^3^ Department of Chemical and Biomolecular Engineering and KI for the Nano Century Korea Advanced Institute of Science and Technology (KAIST) Daejeon 305‐701 Republic of Korea; ^4^ Department of Kinesiology and Community Health Beckman Institute for Advanced Science and Technology University of Illinois at Urbana–Champaign Urbana IL 61801 USA; ^5^ Carl R. Woese Institute for Genomic Biology and Beckman Institute for Advanced Science and Technology University of Illinois at Urbana–Champaign Urbana IL 61801 USA; ^6^ Carle Illinois College of Medicine University of Illinois at Urbana–Champaign Urbana IL 61801 USA; ^7^ Department of Chemical and Biomolecular Engineering University of Illinois at Urbana–Champaign Urbana IL 61801 USA

**Keywords:** acetylcholine receptors, motor neurons, myotubes, neural innervation, neuromuscular junctions

## Abstract

Recreation of a muscle that can be controlled by the nervous system would provide a major breakthrough for treatments of injury and diseases. However, the underlying basis of how neuron–muscle interfaces are formed is still not understood sufficiently. Here, it is hypothesized that substrate topography regulates neural innervation and synaptic transmission by mediating the cross‐talk between neurons and muscles. This hypothesis is examined by differentiating neural stem cells on the myotubes, formed on the substrate with controlled groove width. The substrate with the groove width of 1600 nm, a similar size to the myofibril diameter, serves to produce larger and aligned myotubes than the flat substrate. The myotubes formed on the grooved substrate display increases in the acetylcholine receptor expression. Reciprocally, motor neuron progenitor cells differentiated from neural stem cells innervate the larger and aligned myotubes more actively than randomly oriented myotubes. As a consequence, mature and aligned myotubes respond to glutamate (i.e., an excitatory neurotransmitter) and curare (i.e., a neuromuscular antagonist) more rapidly and homogeneously than randomly oriented myotubes. The results of this study will be broadly useful for improving the quality of engineered muscle used in a series of applications including drug screening, regeneration therapies, and biological machinery assembly.

## 1 Introduction

Skeletal muscle injuries characterized with strain and contusion often lead to significant reduction in the mass and strength of skeletal muscle. Without proper treatments, these pathological conditions may lead to discomfort, pain, disability, and ultimately death. In many cases, the muscular impairment accompanies regression and limited self‐regeneration of neuromuscular junctions. The neuromuscular junction is a specialized synapse at the junction of the motor neuron and myofiber, a critical site that supports neural transmitter release and subsequent regulation of muscle contraction.^1^, ^2^ Morbid or dysfunctional neuromuscular junction causes a series of neuromuscular disorders and diseases.^3^, ^4^, ^5^ Thus, the formation and impairment of the neuromuscular junction have been extensively studied, largely through in vivo studies.

Recently, engineering a muscle tissue innervated by neurons has gained attention because the system potentially allows physiological studies of both normal and impaired tissues at varied length scales. Moreover, the engineered neuromuscular junction could be useful for screening newly developed medicine related to muscular disorders and creating stimulatory devices.^6^, ^7^ The neuromuscular junctions are commonly reproduced by coculturing the skeletal myoblasts and neuronal cells.^7^, ^8^, ^9^, ^10^ For example, a muscle strip cocultured with stem cell‐derived motor neurons exhibits a contraction profile by a natural neurotransmitter (e.g., glutamate) and an antagonist (e.g., curare).^10^ In addition, a microfluidic system was used to spatially organize myotubes and motor neurons and induce neural innervation into the myotubes.^7^ These studies largely focused on evaluating capability of stem cell‐derived neural cells to innervate muscle tissue. Despite these impressive successes, the underlying basis for cross‐talk between muscle and neurons at the neuron–muscle interface is still not sufficiently understood.

Previous studies conducted in vivo suggest that communication between the muscle and motor neurons guide neural innervation.^11^, ^12^ These studies reported that myogenic differentiation in the postsynaptic region can guide synaptogenesis.^11^ For instance, the muscle intrinsically activates muscle‐specific kinase that mediates the expression of acetylcholine receptors (AchRs) that act as a physical pattern at the postsynaptic region. The innervating neurons, on the other hand, secrete agrin proteins that phosphorylates the muscle‐specific kinase and stabilize the clustered acetylcholine receptors in the muscle.^13^, ^14^ The muscle and neurons communicate during these reciprocal events to form neuromuscular junctions. These results imply that a series of intercellular signaling events in the muscle can mediate the neural innervation.

**Figure 1:**
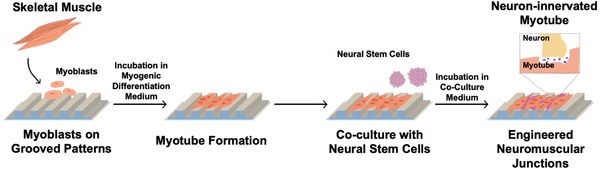
Schematic description of engineering the neuromuscular junction through the sequential coculture of skeletal myoblasts and NSCs on the matrigel‐coated PUA substrates. The substrates were engineered to present grooves with 200, 800, and 1600 nm width.

In this study, we hypothesized that the maturity and alignment of myotubes engineered in vitro would affect the expression of acetylcholine receptors in myotubes and responsiveness of neuron‐innervating muscle to the neurotransmitter and antagonist. First, we cultured primary or C2C12 skeletal myoblasts on the Matrigel‐coated poly(urethane acrylate) (PUA) substrates with grooved patterns (**Figure** **1**). The groove width was varied from 200 nm to 800 and 1600 nm, which encompasses the geometry of collagen fibers in an extracellular matrix (ECM) and myofibrils of the muscle fiber.^15^, ^16^ A flat PUA substrate was used as a control. Next, on the preformed myotubes, we plated neural stem cells (NSCs) and differentiated them into motor neuron progenitor cells. We examined the differentiation lineage and angular alignment of NSCs via immunofluorescence imaging. Last, the neural innervation into myotubes was evaluated by immunofluorescently staining the myosin heavy chain (MHC), acetylcholine receptor, and presynaptic ends of the neurons. The physiological function of the neuromuscular junction was also assessed by measuring the contraction frequency upon exposure to an excitatory neurotransmitter (i.e., glutamate) and a neuromuscular antagonist (i.e., curare).^9^

## 2 Results and Discussion

### 2.1 Engineering Myotubes on the Grooved Substrates

**Figure 2:**
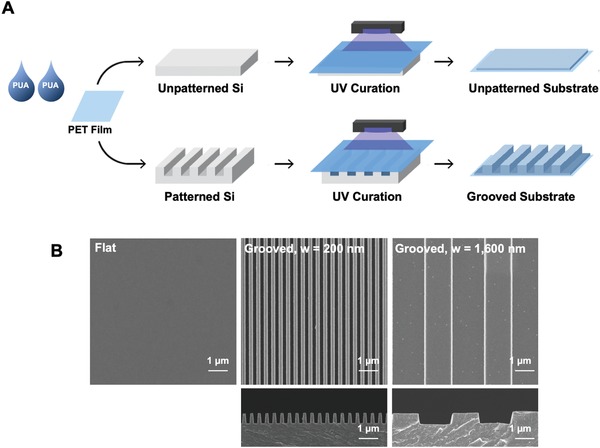
Fabrication procedure and analysis of the grooved PUA substrates. A) Schematic description of the fabrication procedure of the grooved substrates. B) Scanning electron microscope images of the flat PUA substrate, PUA substrate with 200 nm groove width (*w*) and PUA substrate with 1600 nm groove width (*w*). Images in the first and second rows represent the top view and the side view of the substrates, respectively.

First, we prepared PUA substrates with controlled patterns of grooves ranging from 200 to 1600 nm. These substrates were fabricated by placing PUA resin and poly (ethylene terephthalate) (PET) film on the silicon molds as previously described (**Figure** **2**A).^17^ The topographical feature of the pattern was confirmed by scanning electron microscopy images (Figure 2B). These grooved substrates were inspired by the structure of muscle.^17^ The cross‐sectional diameter of a myofibril is around 1 m.^18^ In contrast, collagen fibers, the ECM protein surrounding the skeletal muscle, have cross‐sectional diameters ranging between 200 and 400 nm.^19^

**Figure 3:**
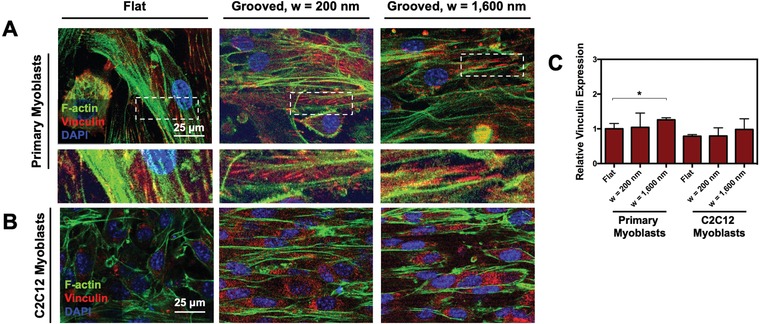
Immunofluorescence staining of F‐actin (green), vinculin (red), and nucleus (blue) of A) primary myoblasts and B) C2C12 myoblasts. The images on the second row of A are magnified views of the area boxed in images. Images were captured 3 d after culture. C) The vinculin expression level quantified with the immunofluorescence images. Each condition was normalized to the vinculin expression value of primary myoblasts adhered to the flat substrate. * represents the statistical significance of the difference of the values between conditions noted in brackets (*n* = 4, *p < 0.05).

We investigated if the grooved substrates modulate the organization of cytoskeletal actin filaments and focal adhesion proteins of the primary and C2C12 myoblasts (**Figure** **3**). According to the immunofluorescence images of F‐actin, the F‐actin filaments of primary myoblasts aligned anisotropically on the grooved substrates, while the orientation was random on a flat substrate (Figure 3A,B). Vinculin was elongated in parallel with the linear pattern of the grooves in the immunofluorescence images. The linear orientation and elongation in the morphology of the focal adhesion complex may contribute to enhanced myogenic differentiation and muscle function after myoblasts differentiate into myotubes.^20^, ^21^ In addition, the primary myoblasts displayed slightly increased expression levels of vinculin, on the substrate with the groove width of 1600 nm (Figure 3C).

**Figure 4:**
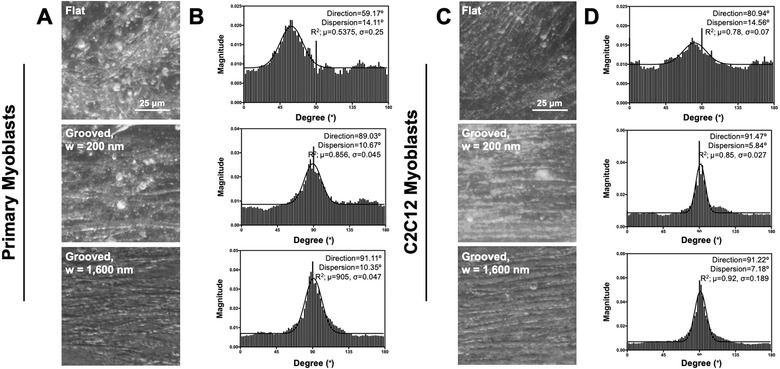
Angular orientation analysis of the skeletal myoblasts. A,B) The primary myoblasts and C,D) C2C12 myoblasts were cultured for 7 d on the flat substrate, a grooved pattern with 200 nm width, and grooved pattern with 1600 nm width. A,C) Morphology images of the primary myoblasts and C2C12 myoblasts, respectively. B,D) Representative histograms of the orientation of primary myoblasts and C2C12 myoblasts. The average value of goodness of fit (*R* ^2^) is indicated as *µ*, and the standard deviation is indicated as σ (*n* = 4).

The angular orientations of the cells were quantified with the optical images of cells (**Figure** **4**). According to the optical images, both primary and C2C12 myoblasts cultured on the grooved substrates aligned in parallel with the grooves. The cells cultured on the flat substrate were, however, randomly oriented. The angular orientation was plotted from 0° to 180° on a histogram using the Directionality plugin in Image J software. This process yielded the histograms in Figure 4B,D. The *y*‐axis indicates the magnitude of Fourier component and the *x*‐axis shows the degree. A single high peak with a low dispersion value and high goodness of fit in the grooved groups suggest that the cells recognize the grooved patterns and align to each other. The flat group showed high dispersion value with low goodness of fit indicating the random orientation of cells.

**Figure 5:**
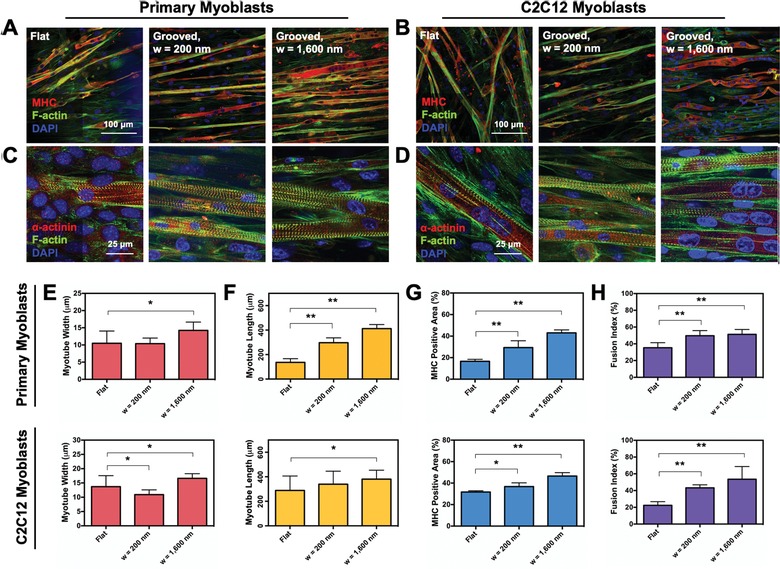
Analysis of the myogenic differentiation of skeletal myoblasts. A,B) Immunofluorescence images of the MHC (red), F‐actin (green), and nucleus (blue) in the differentiated A) primary myoblasts and B) C2C12 myoblasts taken after 10 d of culture in myogenic differentiation medium. C,D) Immunofluorescence images of the sarcomeric‐actinin (red), F‐actin (green), and nucleus (blue) in the differentiated C) primary myoblasts and D) C2C12 myoblasts taken after 10 d of culture in myogenic differentiation medium. E–H) Morphometric analysis of the differentiated skeletal myoblasts based on the immunofluorescence images. The E) myotube width, F) myotube lengths, G) MHC‐positive area, and H) fusion index were quantitatively examined. In each plot, * and ** represent the statistical significance of the difference of the values between conditions noted in brackets (*n* = 4, *p < 0.01, **p < 0.05).

The role of substrate topography on myogenic differentiation level was evaluated by examining the alignment and maturity of the multinucleated myotubes. After 10 d of culture in the myogenic differentiation medium, the myotubes were stained for F‐actin, MHC, and cell nuclei (**Figure** **5**). All three substrates prompted myoblasts to form MHC‐positive myotubes characterized with multinucleation. (Figure 5A,B). As expected, the grooved substrates guided the myotubes to align anisotropically, while myotubes formed on the flat substrate developed in random directions. We further confirmed the myogenic maturation by examining the sarcomeric striation (Figure 5C,D and Figure S1 in the Supporting Information). The myotubes formed with both primary and C2C12 myoblasts cultured on the grooved substrates promoted striation of the myotubes. The relative number of striated myotubes was higher when myoblasts were cultured on the grooved substrates compared to the flat substrate.

With the immunofluorescences images, we performed a morphometric analysis by measuring the width, length, area, and fusion index of MHC‐positive myotubes. These morphometric parameters represent maturity of myotubes. There were significant differences in the size of myotubes between conditions. The myoblasts cultured on the substrate with the groove width of 1600 nm developed MHC‐positive myotubes with the largest width and length (Figure 5E,F). The MHC‐positive area of myotubes was also proportional to the width of the grooves. The dependency was more noticeable with primary myoblasts than C2C12 myoblasts (Figure 5G). The fusion index was quantified by dividing the number of nuclei present in the multinucleated myotubes by the total number of nuclei present (Figure 5H). The fusion index of cells cultured on the grooved substrates was higher than that on the flat substrate, indicating that grooved substrates are advantageous to stimulating mature myotube formation.

We also examined the MHC‐positive myotubes formed on the substrate with the groove width of 800 nm. These myotubes showed minimal differences in the myotube width and area, compared with those formed on the substrate with the groove width of 200 nm (see Figure S2 in the Supporting Information). Therefore, we used substrates with the groove width of 200 and 1600 nm for the following coculture study.

### 2.2 Analysis of Neuronal Differentiation of NSCs on Engineered Myotubes

We studied if the maturity and alignment of myotubes formed on the flat and grooved substrates affect the differentiation lineage of NSCs and the orientation of the differentiated NSCs. We used NSCs because of their potential to differentiate into motor neurons.^10^, ^22^, ^23^ As the first step, we prepared a myotube layer, which covered the flat or grooved substrates entirely. Then, we plated the globular clusters of NSCs, denoted as neurospheres, on the myotubes layer. This allows the NSCs to recognize the orientation of the myotubes instead of the topology of the substrates. Within 3 d, single cells migrated from the neurospheres and adhered on the myotubes.^10^, ^24^

**Figure 6:**
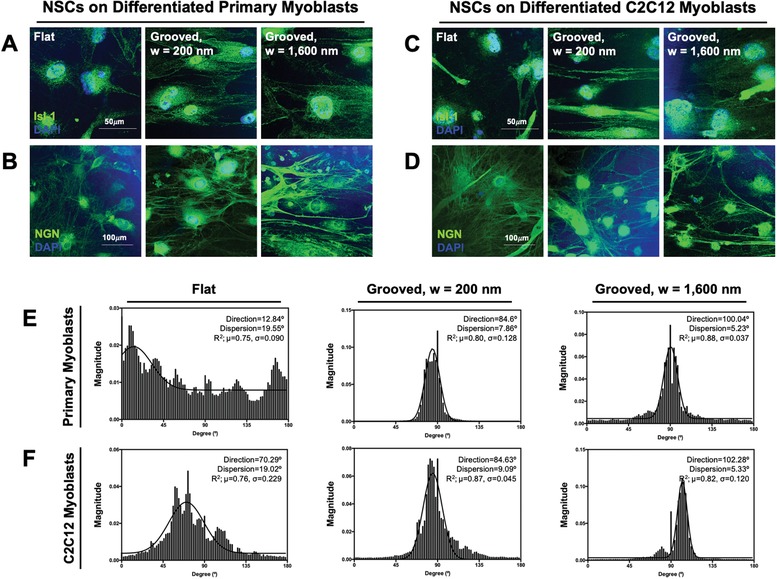
Analysis of the differentiation and alignment of NSCs cultured on the myotubes. Immunofluorescence images of the differentiated NSCs on the myotubes formed with A,B) primary myoblasts and C,D) C2C12 myoblasts. NSCs were stained positively for islet‐1 (Isl‐1, green in panels (A,C), neurogenin‐2 (NGN, green in panels (B,D), and nucleus (blue) after 5 d of culture in the neural differentiation medium. Representative histogram showing the angular orientation of the differentiated NSCs on myotubes formed with E) primary myoblasts and F) C2C12 myoblasts. The average value of goodness of fit (*R* ^2^) is indicated as *µ*, and the standard deviation is indicated as σ (*n* = 4).

The cells migrated from the neurospheres differentiated into motor neuron progenitor cells spontaneously, as confirmed with motor neuron markers, including islet 1 and neurogenin 2 (**Figure** **6**A–D). Islet 1 is a transcription factor essential for differentiating into motor neurons and neurogenin 2 is a transcription factor that specifies motor neuron identity.^25^, ^26^, ^27^, ^28^ The motor neuron progenitor cells positively stained by antibodies to islet 1 and neurogenin 2 stretched their axons in parallel to the myotubes, particularly those formed on the grooved substrate. The motor neuron progenitor cells oriented randomly on the myotubes formed on the flat substrate. The differentiated NSCs were also stained with a neuronal marker, microtubule‐associated protein 2 (MAP2) and a glial marker, glial fibrillary acidic proteins (GFAP). These immunofluorescence images exhibited that the NSCs differentiated into neuronal cells more actively than glial cells when the myotube layer was present (see Figure S3A,B in the Supporting Information). The spontaneous neuronal differentiation of NSCs was observed without the myotubes, as confirmed with positive staining for MAP2, neurofilament (NF), islet 1, and neurogenin 2 (see Figure S3C in the Supporting Information). However, in this condition, differentiated NSCs also expressed GFAP.

The immunofluorescence images of differentiated neurons were analyzed with ImageJ software to determine the angular orientation. Angular orientation was plotted as histograms, and the direction (population mean) and dispersion (standard deviation) were obtained (Figure 6E,F). The neurons cultured on myotubes aligned by the grooved substrates showed two high peaks localized at single peak, with a small dispersion value confirming anisotropic alignment of neurons (Figure 6E,F). The goodness of fit values was above 0.8 indicating that the myoblasts anisotropically aligned better compared to the myoblasts cultured on the flat substrate. Neurons on the myotubes formed on the flat substrates showed a large dispersion value with a low goodness of fit. Thus, the cells were more randomly oriented.

The linear topographical features are known to regulate the differentiation level of NSCs and the spatial organization of the differentiated neurons.^28^, ^29^ The axons of neurons tend to extend more on the linear topography where neurotrophic factors are immobilized.^30^ Apart from these prior studies, this study was conducted by culturing the neurospheres on the myotube layer, which shadowed the substrate pattern. Therefore, this anisotropic alignment of differentiated neurons with myotubes addresses that myotubes can guide the orientation of neurons during this sequential coculture. Moreover, the anisotropically aligned ECM molecules produced by the myotubes, such as collagen, could have guided the orientation of the differentiated neurons. The axons stretching to relatively random directions on the flat substrate support this interpretation.

### 2.3 Morphological Analysis of the Neuron–Muscle Interface

**Figure 7:**
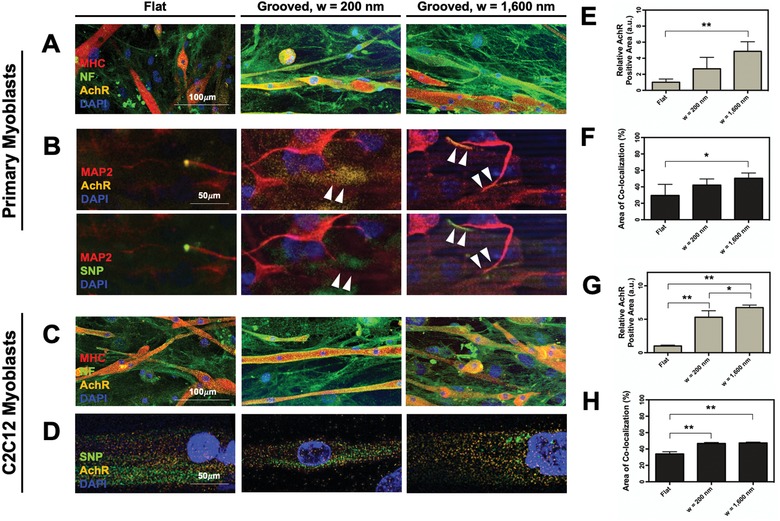
Immunocytochemistry of the neuron‐innervated myotubes. Images were captured after the coculture of primary myoblasts and C2C12 myoblasts with NSCs for 7 d. A,C) Myotubes and neurons stained for MHC (red), NF (green), AchR (orange), and nucleus (blue). B,D) Myotubes and neurons stained for SNP (green), AchRs (orange), and nucleus (blue). B) The motor neuron progenitor cells were additionally labeled with MAP2 (red). E,G) Quantified acetylcholine receptor expression levels (*n* = 4, *p < 0.01, **p < 0.05). The relative acetylcholine receptor expression level was calculated by counting the number of pixels stained positively for acetylcholine receptors in each image and normalizing it to the number obtained with the flat substrate condition. F,H) Average percentage of area where neurofilaments and acetylcholine receptors are colocalized in the myotubes (*n* = 4, *p < 0.01, **p < 0.05). Images of panels (A,B) and graphs of panels (C,D) are the results for primary myoblasts‐derived myotubes. Images of panels (C,D) and graphs of panels (G,H) are the results for C2C12 myoblasts‐derived myotubes.

We examined the mutual interaction between the myotubes and neurons by examining the acetylcholine receptor expression on the myotubes and the neural innervation. Again, myoblasts were cultured on the grooved substrates to form mature myotubes, and neurospheres were plated on the myotubes subsequently. The acetylcholine receptors, MHC, and neurofilaments of differentiated neurons were visualized via immunostaining. On the flat substrate, myotubes and neurons were extended in random directions, as shown with isotropic orientations of the MHCs and neurofilaments (**Figures** **7**A,C). The myotubes on the grooved substrates aligned together with the differentiated neurons, as displayed with the same orientations of MHCs and neurofilaments. Moreover, myotubes formed on the substrates with the groove width of 1600 nm presented larger number of acetylcholine receptors than those formed on the flat substrate, particularly with myotubes formed with primary myoblasts (Images in the first rows of Figure 7A,C). Then, we visualized synaptophysin (SNP) (presynaptic marker) and acetylcholine receptor (postsynaptic marker) to locate the sites where neurons innervate (Figure 7B,D). All conditions showed synaptophysin‐positive nerve ends on myotubes. To confirm the nerve ends, differentiated NSCs were additionally stained with MAP2 for the primary myoblast condition.

Based on the immunofluorescence images, we quantified the relative acetylcholine receptor positive area per myotube in the immunofluorescence image and the area of colocalization of the acetylcholine receptors, MHC, and neurofilaments per myotube. According to the quantitative analysis with immunofluorescence images, myotubes developed on the grooved substrates expressed a higher level of acetylcholine receptors than those on the flat substrate (Figure 7E). Increasing the groove width from 200 to 1600 nm led to a 1.7‐fold increase in the acetylcholine receptors expression level in primary myoblast‐derived myotubes. With C2C12 myoblast‐derived myotubes, the substrate with the groove width of 1600 nm led to the fivefold higher acetylcholine receptor expression than the flat substrate (Figure 7G).

Likewise, primary myoblast‐derived myotubes on the substrate with the groove width of 1600 nm showed a nearly twofold higher percentage of area colocalized by acetylcholine receptors and neurofilaments than those formed on the flat substrate (Figure 7F). This result implies that an increased number of myotubes were innervated by motor neuron progenitor cells on the grooved substrate. The myotubes prepared with C2C12 myoblasts showed a similar trend (Figure 7H). The fluorescence channels were separated to show a clearer expression of each marker (see Figure S5 in the Supporting Information).

### 2.4 Functional Analysis of the Neuron–Muscle Interface

**Figure 8:**
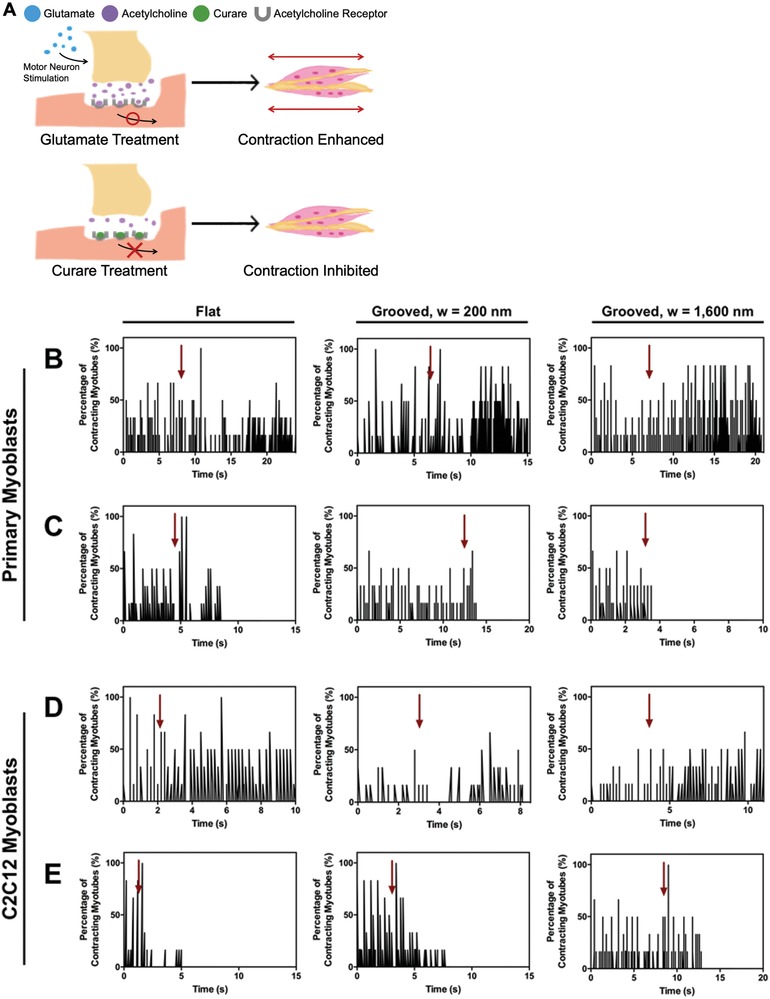
Functionality analysis of the neuron‐innervated myotubes. A) Schematic description of the increased contraction of neuron‐innervated muscle by glutamate and the inhibited contraction by curare. B) Triggered contraction of the primary myoblast‐derived myotubes with the addition of glutamate. C) Inhibited contraction of the primary myoblast‐derived myotubes upon exposure to curare. D) Triggered contraction of the C2C12 myoblast‐derived myotubes with the addition of glutamate. E) Inhibited contraction of the C2C12 myoblast‐derived myotubes upon exposure to curare. In panels (B,D) arrows indicate the time point when glutamate was added. In panels (C,E) arrows indicate the time point when curare was added.

Finally, we evaluated functionality of the motor neuron progenitor cell‐innervated myotubes by recording their response upon exposure to the excitatory neurotransmitter, glutamate, and the neuromuscular junction‐specific antagonist, curare (**Figure** **8**A). Before exposure to glutamate or curare, myotubes showed slightly noticeable spontaneous contraction, regardless of the substrate topography (Figure 8B–E). The myotubes engineered with both primary and C2C12 myoblasts on the substrate with groove width of 1600 nm responded to glutamate and curare more sensitively. The degree of response was specific to cell types. Upon exposure to glutamate, motor neuron progenitor cell‐innervated myotubes on the substrate with groove width of 1600 nm showed higher contraction frequency and number than those formed on the substrate with groove width of 200 nm (Figure 8B and see Table S1 and Movie S1 in the Supporting Information). The myotubes on the flat substrate exhibited an almost imperceptible increase in the contraction frequency.

When the samples were exposed to curare, motor neuron progenitor cell‐innervated myotubes on the substrate with groove width of 1600 nm instantaneously switched off. The motor neuron progenitor cell‐innervated myotubes on the other two substrates showed a lag time ranging from 2 to 5 s before the contraction stopped completely (Figure 8C and see Movie S1 in the Supporting Information).

The difference in muscle contraction between conditions became smaller with myotubes formed with C2C12 myoblasts. Upon exposure to glutamate, there was either proportional increases in the contraction frequency or number with the increase in groove width (Figure 8D and see Table S1 and Movie S1 in the Supporting Information). The muscle contraction was shut down within 5 s for all samples when the samples were exposed to curare (Figure 8E and see Table S1 and Movie S1 in the Supporting Information).

The myotubes formed without NSCs did not show a notable response to either glutamate or curare (see Figure S6 in the Supporting Information). In fact, contraction was either slightly noticeable or not recognizable in the culture. Addition of glutamate to the primary and C2C12 myoblasts did not show significant change in the contraction (see Figure S6A,C in the Supporting Information). Similarly, the myotubes continuously contracted after applying curare to the samples (see Figure S6B,D in the Supporting Information). Because glutamate promotes motor neurons to secrete more acetylcholine molecules from the motor neurons to the skeletal myotubes and enhance contraction, the myotubes without the motor neuron progenitor cells did not respond to glutamate treatment. Curare blocks the acetylcholine receptors in the skeletal myotubes. The blockage of these sites did not have any influence the spontaneous contraction in absence of the motor neuron progenitor cells.

These results address that maturity and orientation of myotubes play important roles in reproducing the neuron‐innervated muscle with an increased sensitivity to glutamate and curare. We suggest that the grooves on the substrates present increased contact area for the myotubes and align the cells anisotropically. In fact, grooves with 200 nm spacing are not wide enough for the cellular membrane to penetrate and form contacts between a substrate and cells, as confirmed with the electron microscopic images (see Figure S4 in the Supporting Information).^31^ On the other hand, myotubes cultured on the substrate with the groove width of 1600 nm protrude their membrane into the grooves. Accordingly, myotubes align with the grooved pattern better than those on the 200 nm grooved substrate. We propose that this enhanced contact with the substrate promotes the anisotropic alignment of myotubes along the grooved pattern.

From the analysis of neuron–muscle interface, we propose that myotubes regulate the lineage of the NSCs and the spatial arrangement of the differentiated motor neuron progenitor cells. The maturity of myotubes influences the acetylcholine receptor expression level, which directly promotes the neuronal differentiation.^32^ The presence of muscle‐derived neurotrophic factors could also contribute to the development and differentiation of NSCs to motor neuron progenitor cells, as maintenance and function of motor neurons depend on secreted factors from the skeletal muscle.^33^, ^34^ Moreover, the aligned myotubes express more acetylcholine receptors, thus, increase the number of neuron‐innervated myotubes as characterized with the immunofluorescence staining of neuron–muscle interface and the response to glutamate and curare. These results confirm that acetylcholine receptors on the myotubes guide and stimulate neural innervation, as previously suggested with the in vivo gene knock‐out studies.^35^, ^36^, ^37^, ^38^ Likely, the agrin molecules secreted by innervating neurons stimulate the aggregation of acetylcholine receptors, which would be systematically examined in future studies.^13^, ^14^

This study provides crucial insights into engineering muscles, morphologically and functionally similar to the natural muscle. To the best of our knowledge, we have demonstrated that the maturity of muscle promotes the neural innervation by mediating the reciprocity between myotubes and neurons for the first time. As a consequence, myotubes display rapid response time and increased contraction number in response to a neural simulator and inhibitor. We proposed that this finding make important scientific impacts in understanding the homeostasis of the normal muscle and the regeneration of the functional muscle. In the long term, this system may work as a vital component of controlling the stimulus responsive behavior of a “living” biological machinery, emerged as a new generation of an autonomous robotic system.^39^, ^40^, ^41^

## 3 Conclusion

In conclusion, this study uncovered an important role of the muscular orientation and maturity in neural innervation and the physiological function of neuromuscular junctions. The nanogrooved substrates with proper groove width facilitated the formation of mature and aligned myotubes compared to the flat substrate. The NSCs subsequently plated on the mature and aligned myotubes differentiated into motor neuron progenitor cells and aligned in the same direction of the preformed myotubes. The mature and aligned myotubes formed on the substrate with groove width of 1600 nm raised the acetylcholine receptor expression level and the percentage of area where motor neuron progenitor cells and acetylcholine receptors are colocalized, compared to myotubes formed on the flat substrate. In consequence, the response of neuron‐innervated muscle contraction to glutamate and curare was more evident when myotubes were more aligned and mature. Altogether, results of this study illuminate the reciprocal activity of myotubes and neurons toward the assembly of the physiologically functional muscle. Therefore, these findings would be useful to improving the quality of engineered tissue used for drug screening, muscular disorder treatment, and biological machinery assembly.

## 4 Experimental Section

*Preparation of Nanogrooved Substrates*: The nanogrooved substrates were fabricated with PUA (MINS‐311RM, Minuta Tech, Gyeonggi, Korea) by using the capillary force lithography techniques reported previously.^17^ Two drops of PUA resin were placed at the center of a nanogrooved Si master and subsequently covered with PET film (Skyrol, SKC Co., Ltd., Seoul, Korea). The PUA resin was exposed to ultraviolet light at 20 mW cm^−2^ for 10 s in the UV curing system (Minuta Tech, Gyeonggi, Korea) (≈365 nm). Then, the PET film with the patterned PUA resin was detached from the Si mater and stabilized for 24 h. The substrates were soaked in isopropyl alcohol for 30 min and distilled water for an additional 30 min. The flat PUA substrate was prepared on the smooth Si surface by following the same curing and cleaning procedure.

*Primary Myoblast Isolation and Culture*: Primary myoblasts were obtained according to the Institutional Animal Care and Use Committee (IACUC) at the University of Illinois, Urbana–Champaign, under an approved protocol # 16089. Mice were sacrificed using CO_2_ inhalation followed by cervical dislocation. Sacrificed mice were placed on ethanol and transferred to a sterile culture hood. Hind limb muscles were dissected and minced in a petri dish 1% v/v penicillin/streptomycin (PS) antibiotic in sterile phosphate buffer saline (PBS). Enzyme solution containing 10% w/v pronase, 3.5% w/v collagenase, and 2.5 mm CaCl_2_ was introduced to the slurry mix and allowed to incubate for 1 h at 37 °C with trituration every 10 min. The mixture was then passed through a 70 × 10^−6^ m filter where a volume of inhibition medium (20% v/v fetal bovine serum (FBS) and 1% v/v penicillin/streptomycin in Hanks' balanced salt solution) equal to the volume of enzyme solution used was added. The solution was again passed through a 40 × 10^−6^ m filter. The conical tubes containing the muscle slurry were spun at 350 g for 5 min, and the pellet was resuspended in the growth medium. Cells were preplated on plastic petri dishes for 3 h, after which the unattached cells were plated on Matrigel coated (1% v/v Matrigel in sterile PBS) Petri dishes. Cells were not allowed past 75% confluence during the passage.

*Culture of Primary and C2C12 Myoblasts for Myogenic Differentiation*: The fabricated PUA substrates were sterilized by using 70% ethanol and washed with sterile PBS three times before use. The surfaces were coated with Matrigel (Corning, New York, NY, USA) to allow cell adhesion. For the coating process, 1% v/v Matrigel in PBS was placed on the PUA substrates and incubated for 1 h. The excess solution was removed by washing the substrates with PBS two times. The primary myoblasts or C2C12 myoblasts (American Type Culture Collection, Rockville, MD, USA) were plated on the PUA substrate at a density of 2 × 10^4^ cells cm^−2^. The PUA substrates plated with primary myoblasts were incubated in growth medium consisting of Dulbecco's modified Eagle medium (DMEM)/F12 50:50 (Corning), r‐fibroblast growth factor (FGF), 1% PS (Gibco, Gaithersburg, MD, USA), 20% FBS (Gibco). The cells were incubated in humidified air containing 5% CO_2_ at 37 °C. Separately, the PUA substrate on which C2C12 myoblasts cultured was incubated in DMEM (Gibco) supplemented with 10% FBS (Gibco) and 1% PS. Myogenic differentiation of both primary cells and C2C12 cells was activated by replacing the growth medium with a differentiation medium (DMEM supplemented with 2% horse serum and 1% PS) after 3 d of culture in the growth medium. The cells were incubated in the differentiation medium for additional 7 d.

*Culture for NSCs for Neuronal Differentiation*: NSCs derived from the mouse brain cortex were purchased from R&D Systems (Minneapolis, MN, USA) and expanded in a neurosphere form by following the manufacturer's protocol. In brief, the cells were plated at a density of 5 × 10^4^ cells mL^−1^ and cultured in DMEM/F12 medium supplemented with the N‐2 supplement (R&D Systems), the epidermal growth factor (EGF) (R&D Systems), and the basic recombinant human fibroblast growth factor (bFGF) (R&D Systems). Fresh EGF and bFGF were added every day during expansion. After culturing the neurospheres in growth medium for 5 d, the medium was replaced with the neural differentiation medium (DMEM/F12 medium supplemented with N‐2 supplement (R&D Systems)). The neurospheres were cultured for 3 d to induce neuronal differentiation before initiating the coculture with the myotubes.

*Coculture of the Differentiated Myoblasts and MSC Neurospheres*: Primary and C2C12 myoblasts were seeded on the PUA substrates coated with Matrigel and differentiated as above. Then, NSC neurospheres cultured in the neural differentiation medium for 3 d were collected and placed on the engineered muscle layer. The two cell populations were incubated in the NSC differentiation medium overnight for stabilization of NSCs. On the next day, the medium was replaced with NSC differentiation medium containing 2% horse serum. The horse serum was included in the coculture media to maintain the differentiated myotubes.^42^ The myoblasts and NSCs were maintained in the coculture medium for another 7 d.

*Immunofluorescence Staining of F‐Actin and Vinculin*: Myoblasts cultured on the flat and grooved substrates were stained for F‐actin, vinculin, and 4′‐6‐diamidino‐2‐phenylindole (DAPI) using the Actin Cytoskeleton and Focal Adhesion Staining Kits (FAK100) (Millipore, Bedford, MA, USA). In brief, after 3 d of culture, cells on the PUA substrates were fixed with 4% w/v paraformaldehyde (Sigma, St. Louis, MO, USA) for 15 min and permeablized with 0.1% v/v Triton X‐100 (Sigma) for 5 min. After incubating the cells in 2% goat serum for blocking unspecific bindings, the cells were incubated with primary vinculin antibody for 1 h at room temperature. Then, vinculin was immunofluorescently labeled with secondary antibodies (Alexa Fluor‐594 donkey antimouse immunoglobulin G (IgG); Invitrogen, Carlsbad, CA, USA). For double immunofluorescence staining, fluorescein (FITC)‐conjugated phalloidin included in the kit was applied simultaneously. After incubation for 1 h, the nuclei of the cells were labeled with DAPI (Sigma). The fluorescence signals were collected with a laser scanning confocal microscope (LSM 700, Carl Zeiss, Jena, Germany).

*Analysis of Cellular Orientation*: The morphology images of the myoblasts were taken with an inverted microscope (Leica DMI 4000B, Leica Microsystems, Wetzlar, Germany) after the myoblasts were cultured in growth medium for 3 d and differentiation medium for 7 d. The directionality of the adhered cells was analyzed by Directionality plugin for ImageJ software. First, the morphology images were converted to 8‐bit grayscale images, and these images were processed with Directionality plugin available on the Analyze tab. This plugin derives a histogram that counts the amount of myotubes in each degree from 0° to 180°. A Gaussian fit was calculated from the highest peak in the histogram. Direction of the myotubes in degree (mean) and the dispersion of the myotubes in degree (standard deviation) were derived by the plug in.^43^ Similarly, the alignment of NSCs cultured on the myotubes was analyzed by following the same procedure. Immunofluorescence images of the neurons positively stained for MAP2 were used for this analysis. The goodness of fit *R* ^2^ value was averaged to confirm orientation of the myotubes.

*Immunofluorescence Staining of Myotubes and Morphometric Analysis*: After culturing the myoblasts for 7 d in the myogenic differentiation medium, the cells were stained immunofluorescently. Cells were fixed with 4% w/v paraformaldehyde (Sigma) for 15 min, permeablized with 0.1% v/v Triton X‐100 (Sigma) for 5 min, and incubated in blocking solution for 45 min. After blocking, the cells were incubated with MF‐20 anti‐MHC (1:400) (iT FX, Developmental Studies Hybridoma Bank, The University of Iowa Department of Biology) at 4 °C overnight. Another set of samples were incubated with antisarcomeric‐actinin antibody (Abcam Cambridge, U.K.). On the next day, MHC was labeled with fluorescence‐tagged secondary antibody (Alexa Fluor‐594 goat anti‐mouse IgG (1:500) (Invitrogen)) and sarcomeric‐actinin was labeled with Alexa Fluor‐568 donkey antirabbit IgG (1:500; Invitrogen). Additionally, phalloidin‐Alexa Fluor 488 (1:200; Invitrogen) and DAPI (Sigma) were used to stain F‐actin and nuclei of the cells, respectively. The length and width of the myotubes were quantified by measuring the length and width of the MHC‐stained myotubes present in the immunofluorescence image. The MHC‐positive area was calculated by quantifying the number of pixels. The fusion index was quantified by calculating the ratio of the number of nuclei in the differentiated myoblasts. The samples were imaged with the laser scanning confocal microscope (LSM 700, Carl Zeiss).

*Immunocytochemical Analysis of Differentiated Neurons*: The immunofluorescence staining of NSCs cultured on the myotubes was fixed, permeabilized, and blocked. Then, the samples were incubated with the motor neuron marker, rabbit monoclonal antiislet 1 (1:100; Abcam), and rabbit polyclonal antineurogenin 2 (1:100; Abcam). Islet 1 and neurogenin 2 were labeled with Alexa Fluor‐488 donkey antirabbit IgG (1:500; Invitrogen). Then, another sample set was incubated with mouse monoclonal anti‐GFAP (1:200; Millipore), and rabbit polyclonal anti‐MAP2 (1:200; Abcam) at 4 °C. The GFAP and MAP2 were labeled with Alexa Fluor‐488 donkey antirabbit IgG (1:500; Invitrogen) and Alexa Fluor‐594 donkey antimouse IgG (1:500; Invitrogen), respectively. The nuclei of cells were separately stained with DAPI. The samples were imaged by the confocal microscope (LSM 700, Carl Zeiss).

*Immunocytochemical Analysis of Neuromuscular Junctions*: The neuromuscular junctions were identified with a site where acetylcholine receptors on the MHC‐positive myotubes and the synaptic ends of the neurofilaments were colocalized. The cells were fixed, permeabilized, and blocked. After blocking, one set of samples was incubated with primary antibodies, MF‐20 anti‐MHC (1:400) (iT FX), and neurofilament‐H (neurofilament 200) (1:50; Sigma) at 4 °C. Another set of samples was treated with chicken polyclonal anti‐MAP2 (1:1000; Abcam) and rabbit monoclonal antisynaptophysin (1:250; Thermo Fisher Scientific, Rockford, IL, USA) to label the myotubes and presynaptic ends of the motor neuron progenitor cells. Lastly, cells treated with MF‐20 and neurofilament‐H were incubated with Alexa Fluor‐488 donkey antirabbit IgG (1:500; Invitrogen) and Alexa Fluor‐594 goat antimouse IgG (1:500) (Invitrogen). The samples treated with anti‐MAP2 antibody and synaptophysin were treated with Alexa Fluor‐488 goat antichicken IgG (1:500; Invitrogen) and Alexa Fluor‐568 donkey antirabbit IgG (1:500) (Invitrogen). The cells were also incubated with the α‐Bungarotoxin‐Alexa Fluor‐647 conjugate (1:1000; Invitrogen) to label acetylcholine receptors on the myotubes. Next, the nuclei were stained using DAPI (Sigma). The final samples were observed under a multiphoton confocal microscope (LSM 710, Carl Zeiss).

*Functionality Analysis of Engineered Neuromuscular Junctions*: The engineered neuromuscular junctions on the substrates were treated with a neurotransmitter, glutamate (400M, Sigma), or an antagonist, (+)‐tubocurarine chloride hydrochloride pentahydrate (curare) (50 m, Abcam), to stimulate or stop the muscle contraction, respectively. Videos were taken using Zeiss Axiocam ERc 5s (Carl Zeiss) attached on an inverted microscope at 30 fps and later processed using FIJI and MatLAB. The region of interest was determined by selecting six active regions in the beginning of data acquisition distributed evenly across the entire field of view. The region of interests was loaded unto MATLAB and tracked displacement of myotube periphery. Modulus of displacement was calculated between each phrase using standard distance formula. Contractions were assigned by selecting positive values after calculating the discreet derivatives of the displacement arrays. Contractions were summed for each frame and plotted against time. The analysis for the control group was performed same as above, but the myotubes were treated without the neural stem cells.

*Statistical Analysis*: All statistical analyses performed in this study were conducted using unpaired Student's t‐test with Graph Pad Prism 6.0 (Graph Pad Software Inc., San Diego, CA, USA). Differences were considered significant at a *p*‐value of less than 0.05. *p*‐value smaller than 0.05 was considered statistically significant.

## Conflict of Interest

The authors declare no conflict of interest.

## Supporting information

Supplementary

Supplementary

Supplementary

Supplementary

Supplementary

Supplementary

Supplementary

Supplementary

Supplementary

Supplementary

Supplementary

Supplementary

Supplementary

Supplementary

Supplementary

Supplementary

Supplementary

Supplementary

Supplementary

Supplementary

Supplementary

Supplementary

Supplementary

Supplementary

Supplementary
