# Supplementary material for: Matrix Topography Regulates Synaptic Transmission at the Neuromuscular Junction
Source: Adv Sci (Weinh). 2019 Jan 17;6(6):1801521. doi: 10.1002/advs.201801521 (PMC6425454; doi:10.1002/advs.201801521)
Supplement: Supplementary file 1 — Supplementary [file ADVS-6-1801521-s002.pdf]

Copyright WILEY-VCH Verlag GmbH & Co. KGaA, 69469 Weinheim, Germany, 2018.

Supporting Information

for *Adv. Sci.,* DOI: 10.1002/advs.201801521

Matrix Topography Regulates Synaptic Transmission at the Neuromuscular Junction

*Eunkyung Ko, Seung Jung Yu, Gelson J. Pagan-Diaz, Ziad Mahmassani, Marni D. Boppart, Sung Gap Im, Rashid Bashir, and Hyunjoon Kong**

SUPPORTING INFORMATION

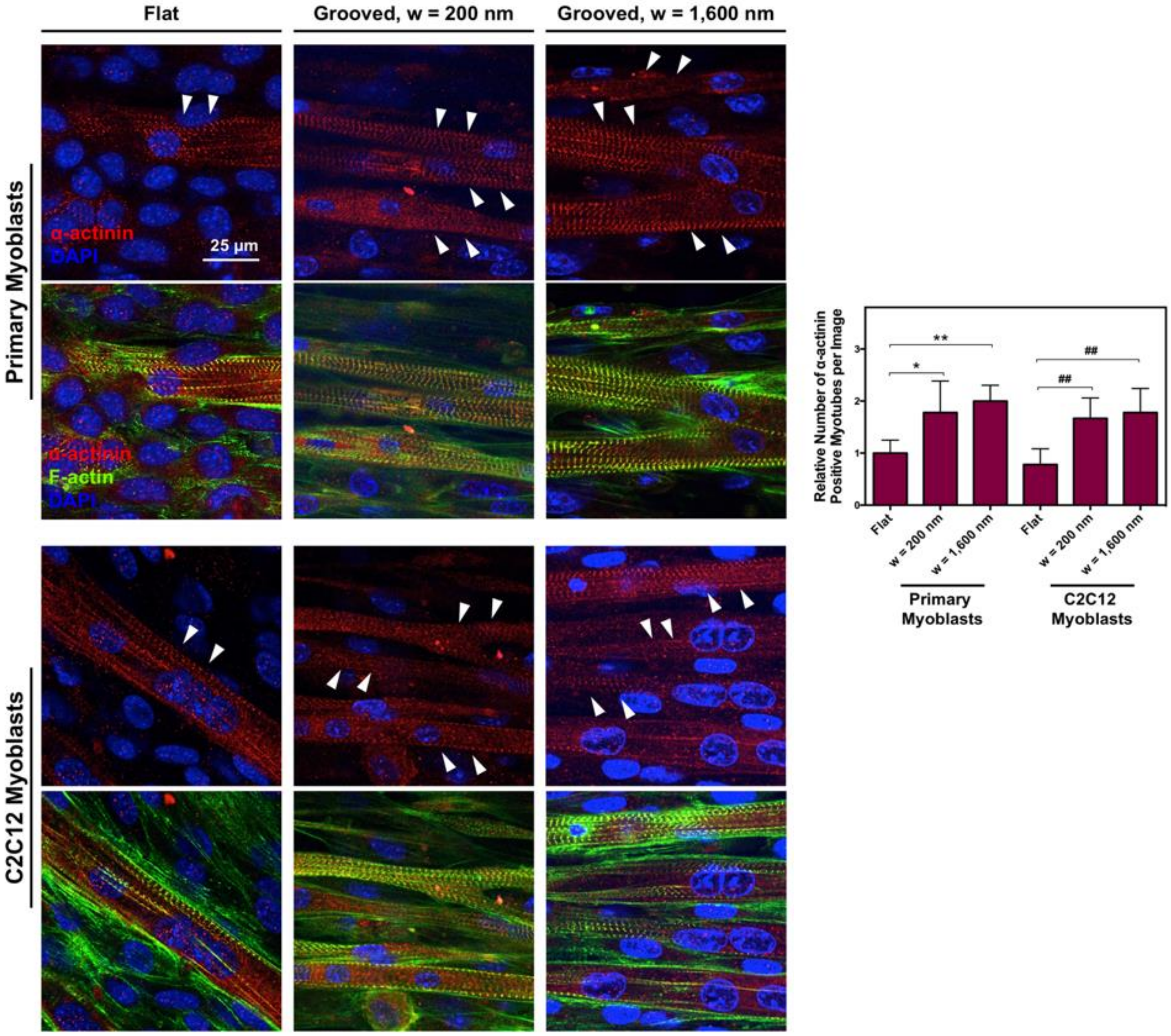

Figure S1. Immunofluorescence staining of sarcomeric α-actinin (red), F-actin (green), and nucleus (blue) of primary myoblasts and C2C12 myoblasts. Images were captured 10 days after culture. The graph represents the relative number of α-actinin positive myotubes per image. * and ** represent the statistical significance of the difference of the values between conditions noted in brackets (n=4, $*p < 0.05$).

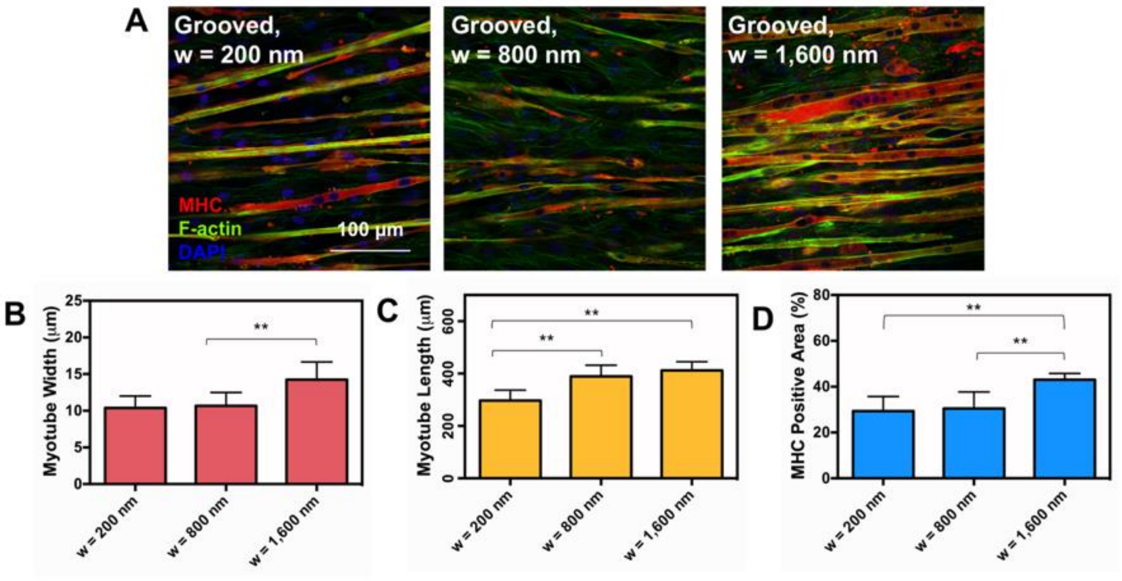

Figure S2. Analysis of the myogenic differentiation of primary skeletal myoblasts cultured on substrates with different groove widths. (A) Immunofluorescence images of the MHC (red), F-actin (green), and nucleus (blue) of myoblasts. Images were taken after 10 days of culture in differentiation medium. (B-E) Morphometric analysis of the differentiated skeletal myoblasts based on the immunofluorescence images. The myotube width (B), myotube lengths (C), MHC-positive area (D) were examined quantitatively. In each plot, * and ** represent the statistical significance of the difference of the values between conditions noted in brackets (n=4, *p < 0.01, **p < 0.05).

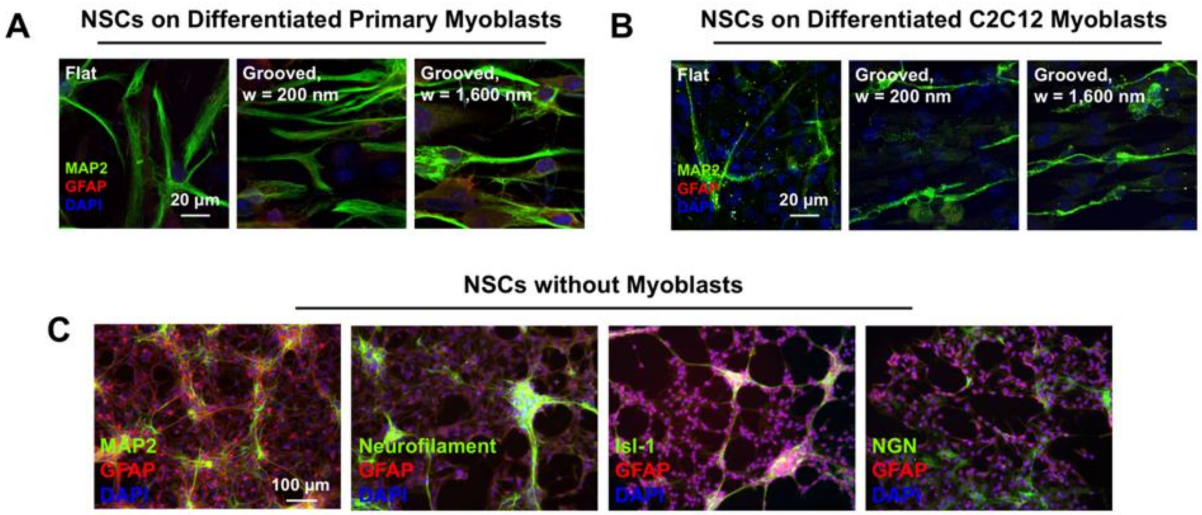

Figure S3. Differentiated NSCs on the myotubes formed with primary myoblasts (A), C2C12 myoblasts (B), and without myoblasts. In (A) and (B), the cells were labeled with MAP2 (green), GFAP (red), and nucleus (blue). In (C), the cells were labeled with MAP2 (green), neurofilament (green), islet-1 (Isl-1, green), GFAP (red), and nucleus (blue) after 5 days of culture in the neural differentiation medium.

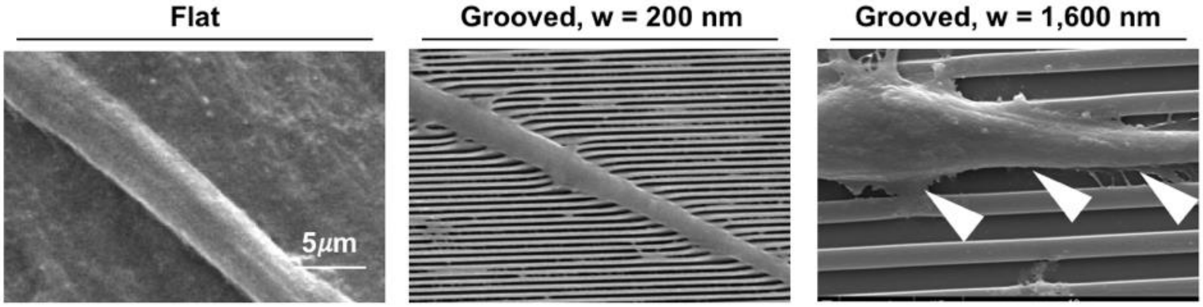

Figure S4. Scanning electron microscope images of the primary myoblasts-derived myotubes on flat and grooved substrates. Arrows indicate the sites where the cell membrane was protruded.

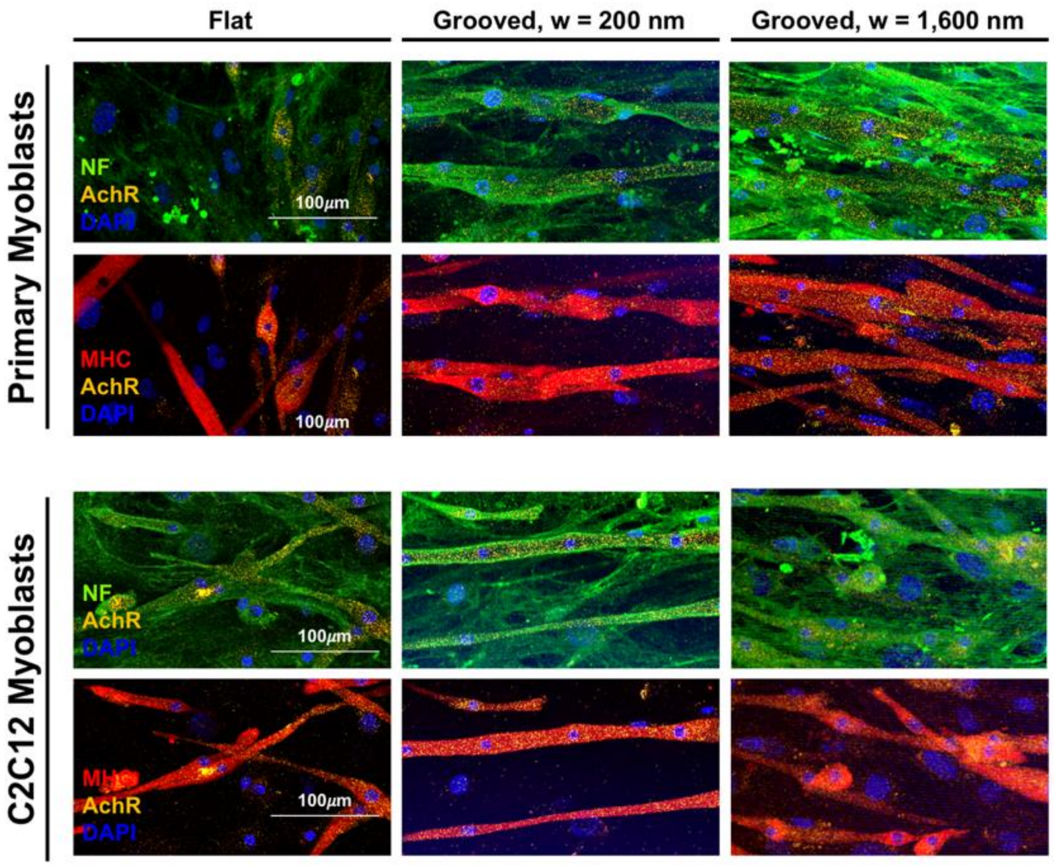

Figure S5. Immunocytochemistry of the neuron-innervated myotubes. Images were captured after the co-culture of primary myoblasts and C2C12 myoblasts with NSCs for 7 days. These images are the same images from Figure 7A-C showing separated channel images.

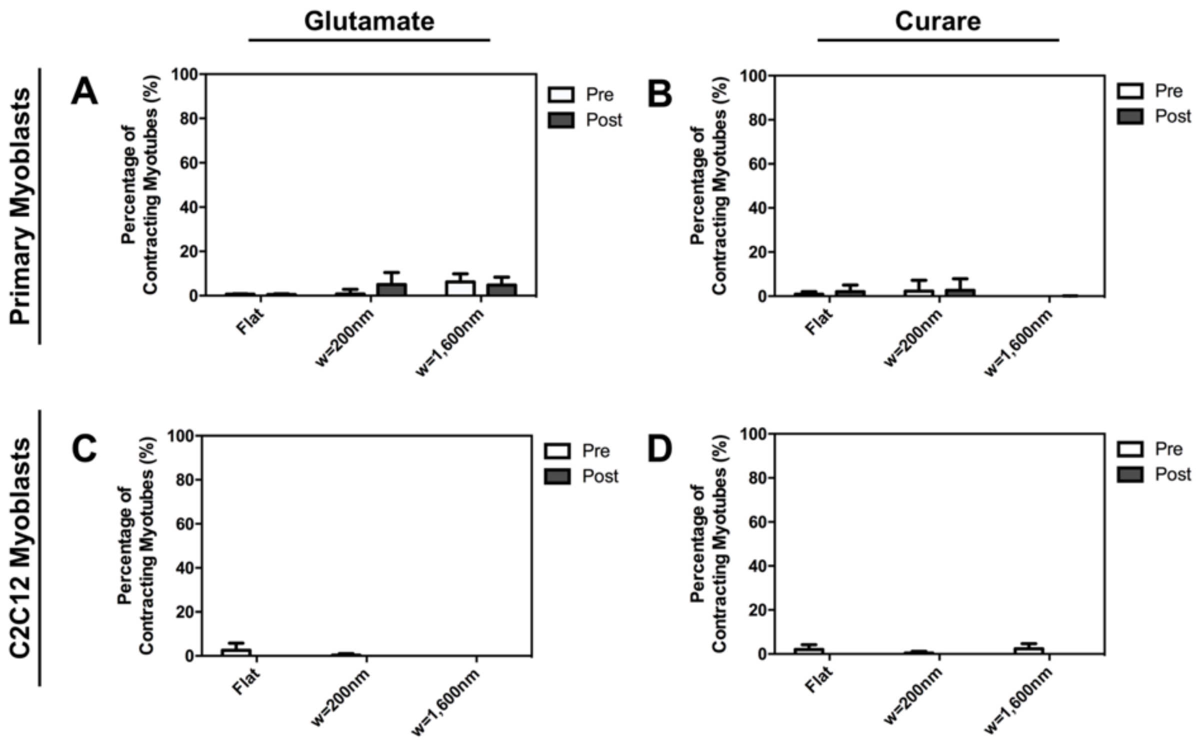

Figure S6. Functionality analysis of myotubes without motor neuron innervation. (A) Contraction change of the primary myoblast-derived myotubes with the addition of glutamate. (B) Contraction change of the primary myoblast-derived myotubes upon exposure to curare. (D) Contraction change of the C2C12 myoblast-derived myotubes with the addition of glutamate. (E) Contraction change of the C2C12 myoblast-derived myotubes upon exposure to curare (n=6).

Figure Number	Cell Type	Groove Width	Added Chemical	Response Time	Result
Figure 8B-Flat-Primary Myoblasts	Primary Myoblasts	Flat	Glutamate	7 seconds	Triggered Contraction
Figure 8B-200-Primary Myoblasts	Primary Myoblasts	200 nm	Glutamate	7 seconds	Triggered Contraction
Figure 8B-1,600-Primary Myoblasts	Primary Myoblasts	1,600 nm	Glutamate	7 seconds	Triggered Contraction
Figure 8C-Flat-Primary Myoblasts	Primary Myoblasts	Flat	Curare	4 seconds	Inhibited Contraction
Figure 8C-200-Primary Myoblasts	Primary Myoblasts	200 nm	Curare	12 seconds	Inhibited Contraction
Figure 8C-1,600-Primary Myoblasts	Primary Myoblasts	1,600 nm	Curare	3 seconds	Inhibited Contraction
Figure 8D-Flat-C2C12 Myoblasts	C2C12 Myoblasts	Flat	Glutamate	2 seconds	Triggered Contraction
Figure 8D-200-C2C12 Myoblasts	C2C12 Myoblasts	200 nm	Glutamate	3 seconds	Triggered Contraction
Figure 8D-1,600-C2C12 Myoblasts	C2C12 Myoblasts	1,600 nm	Glutamate	4 seconds	Triggered Contraction
Figure 8E-Flat-C2C12 Myoblasts	C2C12 Myoblasts	Flat	Curare	2 seconds	Inhibited Contraction
Figure 8E-200-C2C12 Myoblasts	C2C12 Myoblasts	200 nm	Curare	4 seconds	Inhibited Contraction
Figure 8E-1,600-C2C12 Myoblasts	C2C12 Myoblasts	1,600 nm	Curare	8 seconds	Inhibited Contraction

Table 1. Summary of the Supporting Information Movies. Description of the Supporting Information Movies which correspond to the graphs in Figure 8B-E.
